# Association between hyperglycemia and retinopathy of prematurity: a systemic review and meta-analysis

**DOI:** 10.1038/srep09091

**Published:** 2015-03-13

**Authors:** Sunny C. L. Au, Shu-Min Tang, Shi-Song Rong, Li-Jia Chen, Jason C. S. Yam

**Affiliations:** 1 Department of Ophthalmology and Visual Sciences, The Chinese University of Hong Kong, Hong Kong

## Abstract

As the role of hyperglycemia in the development of retinopathy of prematurity (ROP) has not been well established, a meta-analysis of the association between hyperglycemia and ROP was conducted. Studies were identified through literature search in MEDLINE and EMBASE up to June 20, 2014 with keywords related to “hyperglycaemia” and “ROP”. Nine eligible studies involving 1939 neonates with 509 cases of ROP were included. Unadjusted analyses showed that hyperglycemia was significantly associated with ROP (Odds ratio [OR] = 4.16, *P*<0.0001). Comparing with the control, subjects in the ROP group had a significantly longer duration of hyperglycemia (Standardized mean difference [SMD] = 1.21, *P*< 0.0001), and higher mean glucose level. (SMD = 0.88, *P* = 0.0004) However, when combining the adjusted OR (after adjustment for birth weight, gestational age and other factors) provided from individual studies, only borderline significant association were observed on duration of hyperglycemia with ROP (adjusted OR 1.08, *P* = 0.03); and no significant association on mean glucose level with ROP (adjusted OR = 1.08, *P* = 0.15). Hence, hyperglycemia cannot be definitely considered as a risk factor for ROP, and further studies should adjust for potential confounding factors to clarify this association.

Retinopathy of prematurity (ROP) is a preventable cause of childhood blindness. Without treatment, over 45% of eyes can develop permanent visual loss^1^. Other than blindness, ROP also affects visual field, contrast sensitivity, accommodation, convergence and increases risk of strabismus^2^. Non-visual disabilities have also been reported with severe ROP, such as cognitive problems and behavioral problems^3^. The outcome of ROP depends a lot on the standard of neonatal care^4^. Thus, identifying the risk factors for ROP is crucial for prevention and early treatment for the disease.

Most cases of ROP occur in extremely low-gestational-age neonates (gestational age of less than 28 weeks at birth)^5^. Hyperoxia, low birth weight for gestational age, low level and slow increase in post-natal serum IGF-1 concentration are other important risk factors for ROP^6^. ROP can be viewed as an arrest of normal retinal neuronal and vascular development in the preterm infant. Ultimately pathological compensatory mechanisms cause aberrant vascularization of the retina. The more profound the immaturity at birth, and the persistent exposure of the retina to harmful factors, coupled with deficiencies of factors normally provided in utero, the more aggressive is the pathological response^5^.

There is indeed a high prevalence of hyperglycemia, up to more than 50%, in extremely low birth weight (ELBW) infants, particularly during their first week of life^7^. This is mainly related to the associated illnesses (e.g., septicemia), and the response to treatment with corticosteroids^7^,^8^. Garg et al. in 2003 first described the relationship between hyperglycemia and ROP^9^. With the support of the study on rat retinal Muller and mesangial cell suggesting that expression of VEGF protein expression increases in higher glucose concentration^10^,^11^, VEGF was proposed as the link between hyperglycemia and ROP. Since then, many papers have been published with inconsistent results, making the association of hyperglycemia with ROP inconclusive. In an effort to resolve the discrepancy observed across studies, we hereby conduct a systemic review and meta-analysis to evaluate the relationship between ROP and hyperglycemia.

## Methods

### Searching Strategy

Online databases, EMBASE and MEDLINE, were used for electronic search from their starting date to June 20, 2014. The following keywords were used as free words and also as MeSH terms: [“retinopathy of prematurity” OR “ROP” OR “retrolental fibroplasia”] AND [“hyperglycaemia” OR “hyperglycemia” OR “blood glucose” OR “blood sugar” OR “diabetes mellitus” OR “gestational diabetes” OR “DM” OR “GDM”]. All articles and abstracts published in English were identified. The citation lists of relevant articles and reviews were screened to identify additional articles which might have be missed by electronic search. Search strategies were summarized in supplementary table 1.

### Study Selection

The inclusion criteria were defined as (1) original case control studies evaluating the association between ROP and hyperglycemia; (2) hyperglycemia defined as whole blood glucose level greater than 150 mg/dl or 8.5 mmol/l; (3) gestational age of <34 weeks at birth; and (4) raw data provided for calculation. Animal studies, case reports, reviews, abstracts, conference proceedings, editorials, and studies with incomplete data were excluded. Only human studies on clinical aspects of ROP published in English were included.

### Data Extraction

According to the Meta-analysis of Observational Studies in Epidemiology (MOOSE) guidelines for reporting meta-analysis of observational studies^12^, all retrieved records from individual studies were screened and reviewed by two independent investigators (SCLA and SMT). Data were extracted with standardized data sheets. Uncertainties were resolved by consensus with a third investigator (RSS). Data collected included: first author, year of publication, country of study, ethnicity, sample size, gestational age, birth weight, mean blood glucose level in both ROP group and non-ROP group, mean duration of hyperglycemia in both ROP group and non-ROP group, the number of hyperglycemic patients in both ROP group and non-ROP group, adjusted odd ratio (OR) and 95% confidence intervals (CI) of glucose level and duration of hyperglycemia.

According to different approaches of reporting data, and to make comparisons possible, information of hyperglycemia was obtained into 4 forms among different studies: 1) the number of hyperglycemic and non-hyperglycemic subjects in both ROP group and non-ROP; 2) mean blood glucose level in both ROP group and non-ROP group; 3) mean duration of hyperglycemia in both ROP group and non-ROP group; and 4) ORs of blood glucose level and duration of hyperglycemia in multiple logistic regressions analysis of the risk of ROP. Therefore, the association of hyperglycemia and ROP was evaluated via three aspects: 1) overall association of presence of hyperglycemia and ROP; 2) association of duration of hyperglycemia and ROP; and 3) association of mean glucose level of hyperglycemia and ROP.

The unadjusted OR of individual studies for the association of presence of hyperglycemia with ROP was calculated based on the number of hyperglycemic and non-hyperglycemic subjects in both ROP group and non-ROP group. For the association of duration of hyperglycemia with ROP, we analyzed the combined standardized mean difference (SMD) of the duration of hyperglycemia in ROP group versus non-ROP group, and the combined SMD of the mean glucose level of hyperglycemia in ROP group versus non-ROP group^13^. To further evaluate the strength of these associations after adjustment for factors including birth weight and gestational age, we extracted the data of the adjusted OR provided in the studies, and combined the adjusted ORs for analysis. In multiple logistic regression analysis of some studies, the unit of blood glucose is different (10 ml/dl or 1 ml/dl). To unify the unit and combine the ORs, ß (ß = ln(OR)) was calculated, and based on 95% CI = e^(ß-1.96^*^SE)^, the standard error (SE) was determined. To convert the unit of 10 ml/dl to 1 ml/dl, ß and SE were divided by ten and further calculation of ORs and 95% CIs with the unit of 1 ml/dl reversely.

### Quality Assessment

All included studies' qualities were assessed via the Newcastle-Ottawa Scale (NOS)^14^ by two reviewers (SCLA & SMT) independently, and any discrepancy was resolved through consultation with a third reviewer (RSS) Each study was judged on three broad perspectives: the selection of the study groups; the comparability of the groups; and the ascertainment of either the exposure or outcome of interests for case-control or cohort studies respectively. The NOS provides an overall score for methodological quality of up to 9 stars, and a score of 5 or above is considered as satisfactory quality^15^. To assess the comparability, we provided 1 star accounting for oxygen supplement and another for birth weight or gestational age.

### Statistical Analysis

The unadjusted and adjusted odds ratio, standardized mean difference of the duration of hyperglycemia and SMD of mean glucose level in ROP group and control group were synthesized using the fixed- and random-effect models^13^. The Cochran Q statistic testing for heterogeneity across studies and the *I*^*2*^ statistic quantifying the proportion of total variation attributable to between-study heterogeneity were calculated. The Q statistic was considered significant if p<0.1, and *I*^*2*^ above 50% indicated high heterogeneity. If significant heterogeneity was detected, results from the random-effect model was adopted, otherwise, the fixed-effect model^16^. We conducted sensitivity analysis to confirm the association by removing studies with higher risk of introducing bias and to assess the contribution of each study to the heterogeneity by sequentially omitting 1 study and recalculating the combined results. The Modified Egger's regression test were used to assess the potential publication bias^17^, where a value of p<0.05 was considered statistically significant. The Review Manager software (RevMan, version 5.2; the Nordic Cochrane Centre, The Cochrane Collaboration, Copenhagen; 2012) was used for data analysis. The Stata software (version 12; StataCorp LP, College Station, TX) was used to double check the data and conduct the Egger's test. A p value of <0.05 were considered statistically significant.

## Results

### Descriptions of Studies

**Figure 1:**
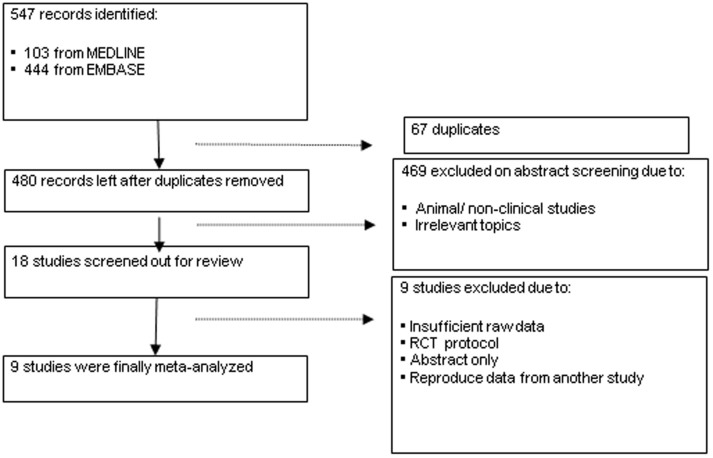
Flowchart of study inclusion.

We identified 103 potential studies through Medline, and 444 through EMBASE. A total of 480 potential studies were identified after elimination of duplicated results. Review of the abstracts excluded 462 studies, for the reasons of being unrelated, animal studies or molecular studies. For the 18 papers screened out, Candel Pau (2012)^18^ was abstracts only, Beardsall (2007)^19^ was a randomized-controlled trial protocol, Beardsall (2008)^20^ reproduced data from another study, and the remaining studies did not give enough primary data for analysis. As a result, 9 studies were eligible and included for meta-analysis, involving 1939 neonates with 509 cases of ROP. (Figure 1) The characteristic of all these 9 studies are summarized in Table 1.

On assessment of the methodological quality using the Newcastle Ottawa Scale, 8 of the 9 studies scored in the range of 7–9, and the remaining one scored 5, which is of satisfactory quality (Supplementary table 2).

### Hyperglycemia and the development of ROP

**Figure 2:**
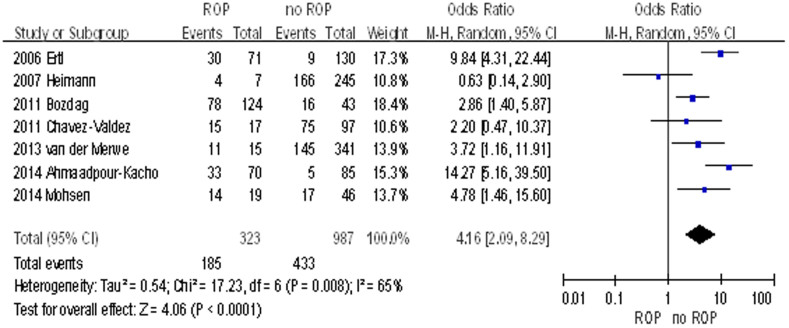
Forrest plot for 7 studies examining odds ratios (ORs) of subjects with hyperglycaemia and without hyperglycaemia in both the ROP and non-ROP group. The bars with squares in the middle represent 95% confidence intervals (95% CIs) and ORs. The central vertical solid line indicates the ORs for null hypothesis.

Seven out of the 9 studies provided primary data on the number of subjects with hyperglycemia and without hyperglycemia in both the ROP and non-ROP groups^21^,^22^,^23^,^24^,^25^,^26^,^27^. Pooling up data from these 7 studies showed that hyperglycemia is associated with ROP with an unadjusted OR of 4.16 (95% CI: 2.09–8.29, I^2^ = 65%, *P*<0.0001) (Figure 2). Sensitivity test further confirm the positive association. There was no publication bias with Egger's test (*P* = 0.403).

### Duration of hyperglycemia and the development of ROP

**Figure 3:**
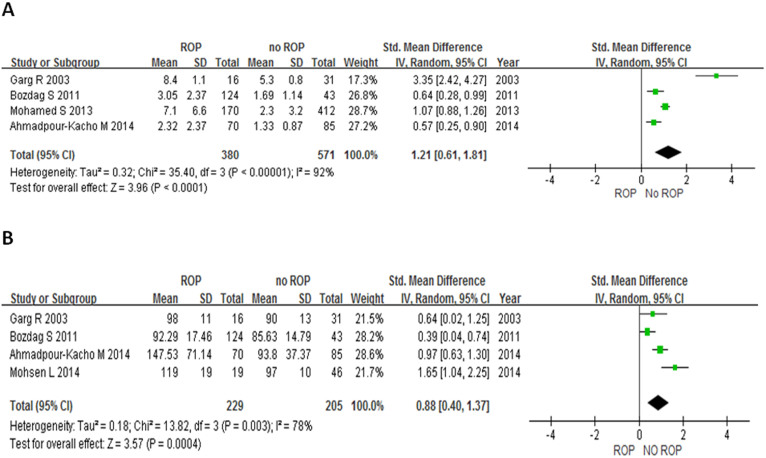
Forrest plot for 4 studies examining standardized mean difference (SMD). The bars with squares in the middle represent 95% confidence intervals (95% CIs) and standardized mean difference (SMD). The central vertical solid line indicates the ORs for null hypothesis. (A). SMD of duration of hyperglycemia in infants with ROP and without ROP. (B). SMD of mean glucose level of hyperglycemia in infants with ROP and without ROP.

Four out of the 9 studies provided the duration of hyperglycemia (in days as the unit of measurement). Pooling up data from these 4 studies showed that duration of hyperglycemia was significantly longer in the ROP group compared to the non-ROP group. (SMD = 1.21, 95% CI: 0.61–1.81, I^2^ = 92%, *P* < 0.0001, Figure 3A).

**Figure 4:**
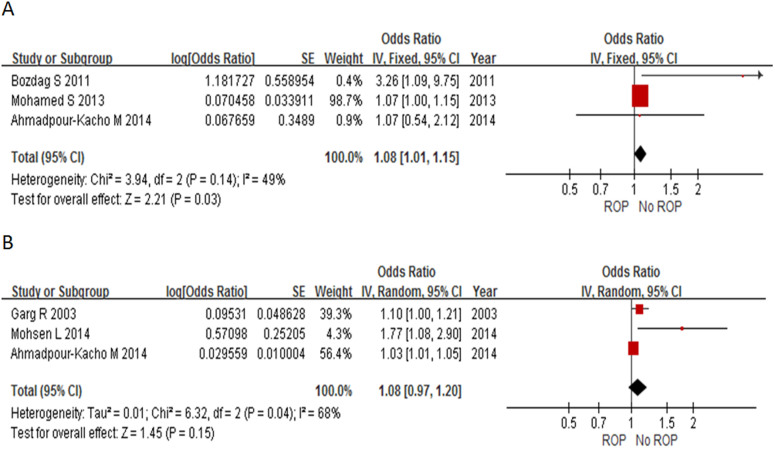
Forrest plot for pooling 3 studies with adjusted OR. The bars with squares in the middle represent 95% confidence intervals (95% CIs) and odds ratios (ORs). The central vertical solid line indicates the ORs for null hypothesis. (A). OR of duration of hyperglycemia. (B). OR of mean glucose level.

In particular, 3 studies provided the adjusted ORs of the association between duration of hyperglycemia and ROP^21^,^25^,^28^. Combining all these adjusted ORs, duration of hyperglycemia showed a borderline association with ROP (adjusted OR = 1.08, 95% CI: 1.01–1.15, I^2^ = 49%, *P* = 0.03, Figure 4A).

### Mean glucose level of hyperglycemia and the development of ROP

Four out of 9 studies provided the mean glucose level of hyperglycemia^9^,^21^,^22^,^25^. Pooling up the data from these 4 studies showed that the mean glucose level was significantly higher in the ROP compared to the non-ROP group (SMD = 0.88, 95% CI: 0.40–1.37, I^2^ = 78%, *P* = 0.0004, Figure 3B).

In particular, 3 studies provided adjusted ORs of the association between the mean glucose level in hyperglycemia and ROP^9^,^21^,^22^. Combining these adjusted ORs revealed that the mean glucose level in hyperglycemia was not associated with ROP (adjusted OR = 1.08, 95% CI: 0.97–1.20, I^2^ = 68%, *P* = 0.15, Figure 4B).

### Sensitivity analysis

We examined the influence of individual studies by omitting 1 study at a time from each of the 2 comparison groups. When we excluded the most influential study (in terms of weight in meta-analysis) of Mohamed et al., the adjusted ORs of duration of hyperglycemia became not significantly associated with ROP (OR = 1.71, 95% CI: 0.58–5.04, I^2^ = 65%, *P* = 0.33) with an increased heterogeneity. On the other hand, the combined adjusted ORs of mean glucose level became significantly associated with ROP (OR = 1.03, 95% CI: 1.01–1.05, I^2^ = 43%, *P* = 0.001) when excluding the study of Mohsen et al. with a reduced heterogeneity. In other analyses, when we conducted the sensitivity analysis, the heterogeneities were reduced (exclude Heimann 2007, Garg 2003 and Mohsen 2014 separately), but all of other results remained unchanged.

### Publication bias

There was no evidence of publication bias in the meta-analysis of the unadjusted OR, SMD of duration of hyperglycemia and SMD of mean glucose level (Egger's test, *P* = 0.403, 0.824 and 0.447, respectively). However, potential publication bias was indicated in the meta-analysis of the adjusted OR of duration of hyperglycemia and adjusted OR of mean glucose level (Egger's test, *P* = 0.026 and *P* = 0.087, respectively), which may be owing to limited number of studies (n = 3).

## Discussion

The meta-analysis of unadjusted data extracted from all studies showed that the OR of developing ROP was significantly higher in infants with hyperglycemia. Further evidence for this association is provided by the statistically significant higher mean glucose level and longer duration of hyperglycemia in the ROP group compared to the control, all based on the unadjusted raw data. However, when analyzing the association by combining the adjusted data provided by each study, only borderline association was observed on the duration of hyperglycemia and ROP, and no association between glucose level and ROP. To our knowledge, this meta-analysis is the first of its kind that analyzes the effect of hyperglycemia on the development of ROP in infants.

Other factors that link hyperglycemia to ROP, including low blood IGF-1 level, use of insulin therapy were not addressed in our study. These 2 factors were studied in clinical applications to show association with ROP^5^. Premature infants are born to have lower IGF-1 as IGF-1 normally increases with gestational age in utero and these premature infants lack the surge of increase when compared to full term infants^5^. IGF-1 is known to counteract insulin resistance and is reduced after preterm birth. Hyperglycemia may be a clinical presentation of low IGF-1 only instead of a real cause of ROP. Whether hyperglycemia has a causal relationship on ROP or just a reflection of illness severity is unknown^29^.

Hyperglycemia as a risk factor can be defined in many ways; also there are many factors that affect the number of measurements to be taken during each individual study, e.g. hematocrit level, use of insulin therapy. Furthermore, different studies have different lengths of study periods, e.g. first 7 days of life^22^,^26^,^27^ vs first month of life^9^,^24^, the longer the study was conducted, the higher the chance of developing hyperglycemia in the subjects.

Heterogeneity was observed across studies in both duration of hyperglycemia and mean glucose level analysis. It is as expected in the mean glucose level analysis that the heterogeneity decreased in the sensitivity test after excluding the least influential studies. As the heterogeneity decreased, we changed it from a random model to a fixed model. Interestingly, in the sensitivity analysis of duration of hyperglycemia, the heterogeneity increased after excluding the most influential study. Therefore, we changed it from a fixed model to a random model. Although changes were observed, the *I*^*2*^ was >40% for all the analysis. This may be explained by the small number of studies in the analysis. In addition, different studies included in the meta-analysis had their results adjusted differently to different known risk factors for ROP, including low birth weight, prematurity, use of oxygen therapy etc., so the adjusted odd ratio is not accurate to compare across different studies.

For the studies included, all have subjects of <2,000 g birth weight. Most were conducted in NICU. Representative results for this low birth weight group of patients can be maintained from the meta-analysis. There should be little discrepancy over the defining criteria for ROP, as most studies listed out the diagnosis of ROP cases according to the International Classification of Retinopathy of Prematurity^30^. For the remaining studies, although diagnosis according to guideline was not mentioned, retina experts or ophthalmologists were involved in the diagnosis.

For studies on hyperglycemia in premature low birth weight neonate, quality assessment by NOS may not be useful in differentiating studies. For “selection”, as most were conducted in NICU on low birth weight neonates, most would have had hospital control. Besides, measurement of blood glucose is actually a fully blinded measurement by the machine. For “exposure/outcome” aspect, reviewing the included studies, only 2 were prospective studies. Therefore loss to follow up should not be a major issue. As a result, the only difference in NOS scores lay on the “comparability” aspect, which was the adjustment of odd ratio to birth weight or low gestational age, and oxygen therapy.

Having too few papers available for our study is a major limitation of our meta-analysis on this topic. For the duration of hyperglycemia and mean glucose level, only 4 studies were eligible for analysis under each category. This caused the heterogeneity and the skewing of results when one study dominated in the weighting.

Our analysis was based mostly on retrospective studies, and thus causation relationship cannot be established.

## Conclusion

Unadjusted analyses showed that hyperglycemia was significantly associated with ROP. However, the association seems to be weakened and even disappeared on analyzing the adjusted OR after adjustment for gestation age, birth weight and other factors. Hence, hyperglycemia cannot be definitely considered as a risk factor for ROP based on current evidence. However, in view of the dearth of studies specifically focusing on the relation between hyperglycemia and ROP, there is a need to conduct further studies on this topic adjusting for potential confounding factors to clarify this association.

## Author Contributions

S.C.L.A. and S.M.T. contributed equally. S.C.L.A. and S.M.T. did the data collection and data analysis; and wrote the main manuscript text and prepared the tables and figures. S.S.R. did the data analysis and critically revised the manuscript. L.J.C. did the data analysis and critically revised the manuscript. J.C.S.Y. conceived the study design, supervised the data collection and data analysis and critically revised the manuscript. All authors reviewed the manuscript.

## Supplementary Material

Supplementary Information Supplementary table 1-3

## Figures and Tables

**Table 1:** Characteristic of studies included for meta-analysis

Year	first author	ROP				Control				Adjusted OR of duration (per day)	Adjusted OR of glucose level (per mg/dl)	Adjusted factors
		Total	NO. of hyperglycemia	Duration (days)	Glucose level (mg/dL)	Total	NO. of hyperglycemia	Duration (days)	Glucose level (mg/dL)			
2014	Ahmadpour-Kacho M	70	33	2.32 ± 2.37	147.53 ± 71.14	85	5	1.33 ± 0.87	93.8 ± 37.37	1.07 (0.54–2.12)	1.03 (1.01–1.05)	gestational age, birth weight transfusion, PO_2_
2014	Mohsen L	19	14	N.A.	119 ± 19	46	17	N.A.	97 ± 10	N.A.	1.77 (1.08–2.86)	day on oxygen, days on respiratiory surpport, gestational age, birth weight, use of breast milk
2013	Mohamed, S.	170	N.A.	7.1 ± 6.6	N.A.	412	N.A.	2.3 ± 3.2	N.A.	1.073 (1.004–1.146)	N.A.	gestational age, ventilator days
2013	Van der Merwe, S. K.	15	11	N.A.	N.A.	341	145	N.A.	N.A.	N.A.	N.A.	N.A.
2011	Chavez-Valdez, R.	17	15	N.A.	N.A.	97	75	N.A.	N.A.	N.A.	N.A.	N.A.
2011	Bozdag, S.	124	78	3.05 ± 2.37	92.29 ± 17.46	43	16	1.69 ± 1.14	85.63 ± 14.79	3.26 (1.09–9.8)	N.A.	birth weight, oxygen, respiratory distress syndrome
2007	Heimann, K.	7	4	N.A.	N.A.	245	166	N.A.	N.A.	N.A.	N.A.	N.A.
2006	Ertl, T.	71	30	N.A.	N.A.	130	9	N.A.	N.A.	N.A.	N.A.	N.A.
2003	Garg, R.	16	N.A.	8.4 ± 1.1	98 ± 11	31	N.A.	5.3 ± 0.8	90 ± 13	N.A.	1.10 (1.00–1.219)	FiO_2_, Vitamin E supplementation

ROP: Retinopathy of Prematurity.

Duration: Duration of hyperglycemia.

N.A.: not available.

## References

[b1] Faia L. J. & Trese M. T. Retinopathy of Prematurity Care: Screening to Vitrectomy. Int Ophthalmol Clin. 51, 1–16 (2011). 21139474 10.1097/IIO.0b013e3182011033

[b2] Holmström G. & Larsson E. Outcome of Retinopathy of Prematurity. Clin Perinatol. 40, 311–321 (2013). 23719312 10.1016/j.clp.2013.02.008

[b3] Schmidt B., Davis P. G., Asztalos E. V., Solimano A. & Roberts R. S. Association Between Severe Retinopathy of Prematurity and Nonvisual Disabilities at Age 5 Years. JAMA 311, 523–525 (2014). 24496539 10.1001/jama.2013.282153

[b4] Gilbert C. Changing challenges in the control of blindness in children. Eye. 21, 1338–1343 (2007). 17914437 10.1038/sj.eye.6702841

[b5] Hellstrom A., Smith L. E. H. & Dammann O. Retinopathy of prematurity. Lancet. 382, 1445–1457 (2013). 23782686 10.1016/S0140-6736(13)60178-6 PMC4389630

[b6] Rivera J. C. *et al.* Understanding retinopathy of prematurity: update on pathogenesis. Neonatology. 100, 343–353 (2011) 21968165 10.1159/000330174

[b7] Hays S. P., Smith E. O. B. & Sunehag A. L. Hyperglycemia is a risk factor for early death and morbidity in extremely low birth-weight infants. Pediatrics. 118, 1811–1818 (2006). 17079549 10.1542/peds.2006-0628

[b8] Lilien L. D., Rosenfield R. L., Baccaro M. M. & Pildes R. S. Hyperglycemia in stressed small premature neonates. J Pediatr. 94, 454–459 (1979). 423036 10.1016/s0022-3476(79)80601-0

[b9] Garg R., Agthe A. G., Donohue P. K. & Lehmann C. U. Hyperglycemia and retinopathy of prematurity in very low birth weight infants. J Perinatol. 23, 186–194 (2003). 12732854 10.1038/sj.jp.7210879

[b10] Brooks S. E., Gu X., Kaufmann P. M., Marcus D. M. & Caldwell R. B. Modulation of VEGF production by pH and glucose in retinal Muller cells. Curr Eye Res 17, 875–882 (1998). 9746434 10.1076/ceyr.17.9.875.5134

[b11] Kim N. H., Jung H. H., Cha D. R. & Choi D. S. Expression of vascular endothelial growth factor in response to high glucose in rat mesangial cells. J Endocrinol. 165, 617–624 (2000). 10828845 10.1677/joe.0.1650617

[b12] Stroup D. F. *et al.* Meta-analysis of observational studies in epidemiology: a proposal for reporting. Meta-analysis of Observational Studies in Epidemiology (MOOSE) group. JAMA. 283, 2008–2012 (2000). 10789670 10.1001/jama.283.15.2008

[b13] Higgins J. P. T. & Green S. (editors). Cochrane Handbook for Systematic Reviews of Interventions Version 5.1.0 [updated March 2011]. The Cochrane Collaboration, 2011. Available from www.cochrane-handbook.org (Date of access: 4^th^ July 2014)

[b14] Wells G. *et al.* The Newcastle-Ottawa Scale (NOS) for assessing the quality of nonrandomised studies in meta-analyses. (2011) Available at: http://www.ohri.ca/programs/clinical_epidemiology/oxford.asp (Date of access: 4^th^ July 2014)

[b15] Kwon B. K., Roffey D. M., Bishop P. B., Dagenais S. & Wai E. K. Systematic review: occupational physical activity and low back pain. Occup Med (Lond) 61, 541–548 (2011). 21727180 10.1093/occmed/kqr092

[b16] DerSimonian R. & Laird N. Meta-analysis in clinical trials. Control Clin Trials. 7, 177–188 (1986). 3802833 10.1016/0197-2456(86)90046-2

[b17] Harbord R. M., Egger M. & Sterne J. A. A modified test for small-study effects in meta-analyses of controlled trials with binary endpoints. Stat Med. 25, 3443–3457 (2006). 16345038 10.1002/sim.2380

[b18] Candel Pau J. *et al.* Perinatal outcome of preterm intrauterine-growth-restricted infants. J Matern Fetal Neonatal Med. 25, 111 (2012).22958037

[b19] Beardsall K. *et al.* A randomised controlled trial of early insulin therapy in very low birth weight infants, “NIRTURE” (neonatal insulin replacement therapy in Europe). BMC Pediatr. 7, 29 (2007). 17692117 10.1186/1471-2431-7-29 PMC1994677

[b20] Beardsall K. & Dunger D. Insulin therapy in preterm newborns. Early Human Development. 84, 839–842 (2008). 18848411 10.1016/j.earlhumdev.2008.09.013

[b21] Ahmadpour-Kacho M. *et al.* Correlation between hyperglycemia and retinopathy of prematurity. Pediatr Int. 56, 726–730 (2014). 24803073 10.1111/ped.12371

[b22] Mohsen L. *et al.* A prospective study on hyperglycemia and retinopathy of prematurity. J Perinatol. 34, 453–457 (2014). 24674983 10.1038/jp.2014.49

[b23] Van der Merwe S. K., Freeman N., Bekker A., Harvey J. & Smith J. Prevalence of and risk factors for retinopathy of prematurity in a cohort of preterm infants treated exclusively with non-invasive ventilation in the first week after birth. S Afr Med J. 103, 96–100 (2013). 23374319 10.7196/samj.6131

[b24] Chavez-Valdez R., McGowan J., Cannon E. & Lehmann C. U. Contribution of early glycemic status in the development of severe retinopathy of prematurity in a cohort of ELBW infants. J Perinatol. 31, 749–756 (2011). 21415837 10.1038/jp.2011.19

[b25] Bozdag S. *et al.* Serum fructosamine and retinopathy of prematurity. Indian J Pediatr. 78, 1503–1509 (2011). 21732016 10.1007/s12098-011-0515-9

[b26] HeimannK. *et al.* Are recurrent hyperglycemic episodes and median blood glucose level a prognostic factor for increased morbidity and mortality in premature infants 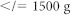 ? J Perinat Med. 35, 245–248 (2007).1748015510.1515/JPM.2007.057

[b27] Ertl T., Gyarmati J., Gaal V. & Szabo I. Relationship between hyperglycemia and retinopathy of prematurity in very low birth weight infants. Biol Neonate. 89, 56–59 (2006). 16155387 10.1159/000088199

[b28] Mohamed S., Murray J. C., Dagle J. M. & Colaizy T. Hyperglycemia as a risk factor for the development of retinopathy of prematurity. BMC Pediatr. 13, 78. (2013). 23679669 10.1186/1471-2431-13-78 PMC3689099

[b29] Hey E. Hyperglycaemia and the very preterm baby. Semin Fetal Neonatal Med. 10, 377–387 (2005). 15927546 10.1016/j.siny.2005.04.008

[b30] International Committee for the Classification of Retinopathy of Prematurity. The International Classification of Retinopathy of Prematurity revisited. Arch Ophthalmol. 123, 991–999 (2005). 16009843 10.1001/archopht.123.7.991

