# Supplementary material for: Association between hyperglycemia and retinopathy of prematurity: a systemic review and meta-analysis
Source: Sci Rep. 2015 Mar 13;5:9091. doi: 10.1038/srep09091 (PMC4357868; doi:10.1038/srep09091)
Supplement: Supplementary Information — Supplementary table 1-3 [file srep09091-s1.pdf]

Manuscript Title: Association between hyperglycaemia and retinopathy of prematurity: a systemic review and meta-analysis

Author lists: Sunny C. L. Au ; Shu-Min Tang; Shi-Song Rong;Li-Jia Chen; Jason C.S. Yam

Supplementary Table 1. Searching Strategy

1. exp retrolental fibroplasia/
2. retinopathy of prematurity.mp.
3. ROP.mp.
4. 1 or 2 or 3
5. exp hyperglycemia/
6. exp glucose blood level/
7. blood sugar.mp.
8. exp pregnancy diabetes mellitus/
9. gestational diabetes mellitus.mp.
10. GDM.mp.
11. exp diabetes mellitus/
12. DM.mp.
13. 5 or 6 or 7 or 8 or 9 or 10 or 11 or 12
14. 4 and 13

Supplementary table 2. Assessment of the Methodological Quality of the Studies Using Newcastle-Ottawa Scale

AUTHOR	SELECTION	COMPARABILITY	OUTCOME/EXPOSURE	SCORE
Garg 2003	****	**	***	9
Ertl 2006	****	*	***	8
Heimann 2007	***	*	***	7
Bozdag 2011	****	**	***	9
Chavez-Valdez 2011	***		**	5
Mohamed 2013	****	**	***	9
Van der Merwe 2013	****		***	7
Mohsen 2014	***	**	**	7
Ahmadpour-Kacho 2014	****	*	***	8

Supplementary table 3. Summary of the Clinical Profile of Infants and Methodological Characteristics of the studies

Year of Publication	First Author	Study Design	Study location	Defination and Grading of ROP	Definition of Hyperglycemia	Gesational Age of ROP (week)	Gestational Age of Non-ROP (week)	Birth Weight of ROP	Birth Weight of Non-ROP
2014	Ahmadpour-Kacho M.	case-control study	Iran	international classification of ROP	>150mg/dL	29.91 ± 2.46	30.59 ± 1.97	1238.57g ± 344.77	1327.53g ± 293.03
2014	Mohsen L	prospective cohort study	Egypt	international classification of ROP	>150mg/dL	30 ± 1	31.5 ± 0.9	1227g ± 204	1450g ± 202
2013	van der Merwe, S. K.	case-control study	South Africa	threaten sight	>8.5 mmol/l	27.3 ± 3.1	28.4 ± 1.8	851g ± 230.5	949.3g ± 177.6
2013	Mohamed, S.	case-control study	USA	international classification of ROP	>150mg/dL	30 ± 1	31.5 ± 0.9	1227g ± 204	1450g ± 202
2011	Chavez-Valdez, R.	case-control study	USA	international classification of ROP	>150 mg/dL	26 ± 1.5	26.8 ± 2	772g ± 124	793g ± 133
2011	Bozdag, S.	case-control study	Turkey	international classification of ROP	>150 mg/dL	28.48 ± 1.94	29.6 ± 1.79	1092g ± 212.9	1269.07g ± 206.6
2007	Heimann, K.	case-control study	Germany	Not mention	>150 mg/dL	Not mentioned	Not mentioned	Not mentioned	Not mentioned
2006	Ertl, T.	case-control study	Hungary	international classification of ROP	≥ 8.5 mmol/L	27.0 ± 1.9	30.1 ± 2.2	971g ± 227	1237g ± 192
2003	Garg, R.	case-control study	USA	international classification of ROP	>150 mg/dL	24.8	25	663g	717g
